# Deletion of IL-18 Expression Ameliorates Spontaneous Kidney Failure in MRL^lpr^ Mice

**DOI:** 10.1371/journal.pone.0140173

**Published:** 2015-10-14

**Authors:** Bastian Schirmer, Dirk Wedekind, Silke Glage, Detlef Neumann

**Affiliations:** 1 Institute of Pharmacology, Hannover Medical School, Hannover, Germany; 2 Institute of Laboratory Animal Science, Hannover Medical School, Hannover, Germany; INSERM-Université Paris-Sud, FRANCE

## Abstract

The role of IL-18 in the pathogenesis of systemic lupus erythematosus is still not definitively solved. In this study, we generated MRL^lpr^ mice, which develop a disease resembling systemic lupus erythematosus, genetically devoid of IL-18 expression. These mice in comparison to IL-18-competent MRL^lpr^ mice show reduced signs of renal pathogenesis, while other parameters such as mean survival time, lymphadenopathy, constitutive interferon-γ production, and frequency of CD3^+^B220^+^ abnormal T cells were without differences. We conclude that in the systemic lupus erythematosus syndrom IL-18 is involved specifically in the renal pathogenesis.

## Introduction

The interleukin (IL)-1 family member IL-18, originally referred to as ‘interferon (IFN)-γ-inducing factor (IGIF)’, is a proinflammatory cytokine [1]. Major cellular targets of IL-18 are T helper (Th) 1 cells, natural killer (NK) cells, and CD8^+^ T cells, in which it increases proliferation, lytic activity and IFNγ production [2–4]. IL-18 by itself does not induce IFN-γ expression, but rather acts synergistically with e.g. IL-12, which induces expression of the IL-18 receptor [5, 6].

The participation of IL-18 in inflammatory diseases including autoimmunity has been demonstrated in several animal models and also in humans [7–9]. Of those, the predominant ones are type 1 diabetes, multiple sclerosis, rheumatoid arthritis, psoriasis, and possibly also systemic lupus erythematosus (SLE). SLE is characterized by hyper-gammaglobulinemia, autoantibody production and immune complex formation, eventually leading to end-organ damage, including vasculitis and fatal renal failure [10]. The disease is associated with genetic polymorphisms in the *il18* gene in diverse populations [11–13]. In plasma of SLE patients the concentration of IL-18 is enhanced and correlates with the disease activity [14–16].

Homozygous MRL/Mp-*Tnfrsf6* ^*lpr/lpr*^ (MRL^lpr^) mice serve as model for human SLE [17]. Th-cells, characterized by the expression of CD4, centrally contribute to the pathogenesis of MRL^lpr^ mice [18]. Of those, IL-17-expressing cells constitute a decisive effector population [19]; however, also Th1- and Th2-derived cytokines are apparently involved: IFN-γ, the prototypical Th1-cytokine, is detected at rather high concentrations in serum of MRL^lpr^ mice and deletion of IFN-γ or IFN-γ receptor expression leads to reduced pathological signs [20–23]. In accordance with these observations, administration of IFN-γ exacerbates the disease [24]. The contribution of IL-18 to the syndrome of MRL^lpr^ mice is still discussed controversially. Lymph node cells or autoreactive T-cell lines obtained from MRL^lpr^ mice are hyper-reactive to IL-18 stimulation due to a constitutive high expression of the IL-18 receptor [25]. Consequently, administration of IL-18 to MRL^lpr^ mice worsened the disease [26] and MRL^lpr^ mice vaccinated against IL-18 demonstrated reduced pathological signs [27, 28]. However, IL-18 administration by itself is unable to induce a lupus-like pathogenesis in MRL/Mp-*Tnfrsf6* ^+/+^ mice [26].

Genetic deletion of the IL-18 receptor in MRL^lpr^ mice has been described in two independent studies with contradictory conclusions [29, 30]. While the first one claimed the amelioration of the lupus-like disease due to the absence of the IL-18 receptor expression [30], the second one could not find any consequences of the deletion [29]. Moreover, there are indications, that the IL-18 receptor can be engaged not only by IL-18 itself, but also by another, yet unidentified ligand [31].

Thus, in the present study, we genetically deleted IL-18 expression in MRL^lpr^ mice in order to specifically analyze the contribution of IL-18 to the lupus-like disease. We demonstrate that IL-18 is involved mainly in the autoimmune lupus nephritis.

## Materials and Methods

### Mice

Homozygous MRL/MpOlaHsd-*Tnfrs6* ^*lpr/lpr*^ mice and MRL/MpOlaHsd (MRL^+/+^) mice were purchased from Harlan/Winkelmann (Borchen, Germany) and housed and bred in the animal facility of the Hannover Medical School. IL-18 deficient MRL^lpr^ mice were obtained by ten generations of backcrossing of the Il18^tm/tmAki^ genotype from C57BL/6 mice (generously provided by S. Akira, Osaka, Japan [32]) onto the MRL^lpr^ strain. Backcrossing was assisted by genetic diagnoses of the *Il18* locus and SNPs (small nuclear polymorphism) analyses discriminating the C57Bl/6 and MRL^lpr^ strains. Resulting strains were homozygous MRL/Mp-*Tnfrs6* ^*lpr*^ *Il18* ^*tm/tm*^ (MRL^lpr^IL18^tm/tm^) and heterozygous MRL/Mp-*Tnfrs6* ^*lpr*^ *Il18* ^*+/tm*^ (MRL^lpr^IL18^+/tm^). Mice were inspected daily and their health and constitution was ranked using a scoring system as detailed in table 1 (Table 1; [33]). Due to the autoimmune lupus-like disease, the mice acquired a moribund state rather quickly; however mice did not die unexpectedly or spontaneously since those which ranked score ≥4 were euthanized by CO_2_ inhalation followed by cervical dislocation. The age of mice when ranking score ≥4 are the bases for calculating their survival times. For all other analyses (except measurement of proteinuria), mice at pre-defined ages anaesthetized by CO_2_ inhalation and killed by exsanguination via cardiac puncture. Otherwise, analgesics and anaesthetics were not used in this study. Experimental procedures were performed according to the German Animal Welfare Act (Tierschutzgesetz, § 4) and approved by the Local Institutional Animal Care and Research Advisory Committee of the Hannover Medical School and the Lower Saxony State Office for Consumer Protection and Food Safety (Approval ID: 2012/8).

**Table 1:** Evaluation of mice’s health.

Score	Activity	Body weight	General state of health	Behavior
**1**	Very active	Unchanged or increased	Pelt smooth, bright; eyes clear, bright; normal temperature	Attentive; fast moving
**2**	Active	Reduced by ≤ 5%	Pelt with defects (reduced care); slightly enhanced temperature	Occasional breaks in movement
**3**	Less active	Reduced by 6% -14%	Pelt dull, disordered; turbid eyes; enhanced muscle tonus; enhanced temperature	Regular breaks in movement; sufficiently reactive to environment
**4**	Barely active	Reduced by 15–19%	Pelt dirty; clotted or watery orifices; bended posture; high muscle tonus; high temperature	Self-isolation; less reactive to environment; activity poor, slow
**5**	Lethargic	Reduced by ≥ 20%	Cramping; paralysis; flat breathing; low temperature	No activity; not reactive to environment

### Serum preparation

The blood obtained by exsanguination was clotted for either one hour at room temperature or overnight at 4°C. Serum was separated by centrifugation at 10,000 g, 4°C for 20 min, aliquoted and stored at– 80°C until use.

### Organ and cell preparation

Axillary and inguinal lymph nodes, spleens, and kidneys were removed and dissociated into single cell suspensions using the gentleMACS dissociator system (Miltenyi Biotech, Bergisch Gladbach, Germany).

### Lymphadenopathy and splenomegaly

Lymphocyte proliferation in lymph nodes and spleen was assessed in parallel with two methods. Weights of lymph nodes and spleens were evaluated routinely to assess the macroscopic lymphadenopathy. In addition, nucleated cell counts were performed after organ dissociation.

### Proteinuria

Urine protein levels were assessed semi-quantitatively using Combur Test strips (Boehringer-Mannheim, Mannheim, Germany). Small volumes (20 μl) of spontaneously voided urine were taken at weekly intervals.

### Determination of renal damage

Renal tissue was either fixed in formalin and embedded in paraffin or was prepared for cryo-conservation. Sections were stained with hematoxylin and eosin (H&E), periodic acid-Schiff (PAS), or silver based reticulin. Kidney lesions were scored according to Table 2, analyzing at least 2 sections of each kidney and 55 +/- 5 glomeruli of each section.

**Table 2:** Evaluation of kidney damage.

Score	Interstitial cell infiltration and vasculitis	Mesangial hyper-cellularity	Protein casts in distal tubuli	Hypercellularity of Bowmann capsules	Sclerotic changes of the mesangium	Lobular accentuation of glomerulopathy	Thickening of basal membrane in glomeruli/ tubuli	Gross inflammatory pathology (% inflamed area)
**0**	no sign	no sign	no sign	no sign	no sign	no sign	no sign	No inflammation
**1**	mild in some areas	mild in some areas	mild in some areas	mild in some areas	mild in some areas	mild in some areas	mild in some areas	minor; 10%
**2**	clear and frequent	clear and frequent	clear and frequent; some dilatation	clear and frequent	clear and frequent; some lumen obstruction	clear and frequent	clear and frequent	mild; 10–20%
**3**	severe and widespread	severe and widespread	severe and widespread, frequent dilatation	severe and widespread	severe and widespread, frequent lumen obstruction	severe and widespread	severe and widespread	moderate; 25–50%
**4**								serious; 50–75%
**5**								severe; > 75%

### Statistical analysis

Where appropriate, data are presented as individual values and the mean ± SD. Statistical significance was determined using Student’s *t* test or one- or two-way ANOVA with Bonferroni’s post-test. Proteinuria data were analyzed by Kruskal-Wallis test with Dunn’s post-test, whereas survival analysis was performed using Kaplan-Meier method and significance of differences in survival was determined using Mantle-Cox and Gehan-Breslow-Wilcoxon tests.

## Results

**Fig 1:**
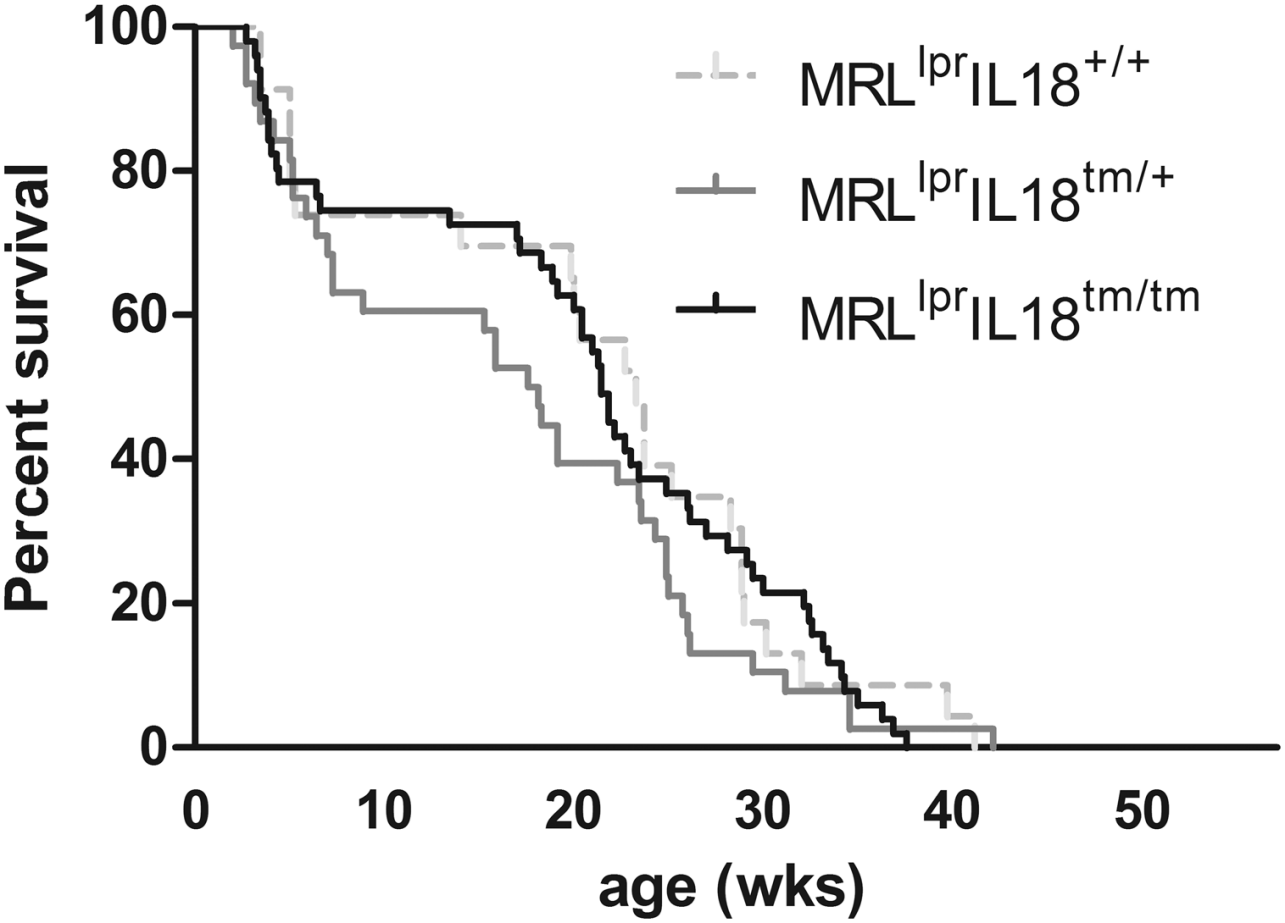
Deletion of IL-18 expression does not affect survival. MRL^lpr^ mice displaying an IL-18 expression status as indicated were housed as described in the methods section. Individual ages (weeks) when they reached a health status ranking score ≥4 (table 1) were documented. Presented are pooled data from n = 23, 38, and 51 individuals of MRL^lpr^, MRL^lpr^Il18^+/tm^, and MRL^lpr^Il18^tm/tm^ mice, respectively. The three data sets were analysed by Log-rank (Mantel-Cox) test and revealed no statistically significant differences.

In order to analyze a possible role of IL-18 in the pathogenesis of the lupus-like disease in MRL/Mp-*Tnfrs6lpr (*MRL^lpr^) mice, we intensively backcrossed the Il18^tm/tmAki^ genotype from C57BL/6 mice onto the MRL^lpr^ strain to obtain MRL/Mp-*Tnfrs6lprIl18* ^*tm/tm*^ (MRL^lpr^IL18^tm/tm^) and MRL/Mp-*Tnfrs6lprIl18* ^*+/tm*^ (MRL^lpr^IL18^+/tm^) mice. Differences in the mean survival time of MRL^lpr^ mice due to the genetic deletion of IL-18 expression (MRL^lpr^: 163 days, MRL^lpr^IL18^+/tm^: 125 days; MRL^lpr^IL18^tm/tm^ 150 days; Fig 1) were statistically not significant.

A major symptom occurring in MRL^lpr^ mice is the eponymous lymphoproliferation, resulting in strongly enlarged secondary lymphoid organs. Analyses of spleen and lymph nodes weights and cell numbers revealed no statistically significant differences between MRL^lpr^ and MRL^lpr^IL18^tm/tm^ mice (data not shown).

**Fig 2:**
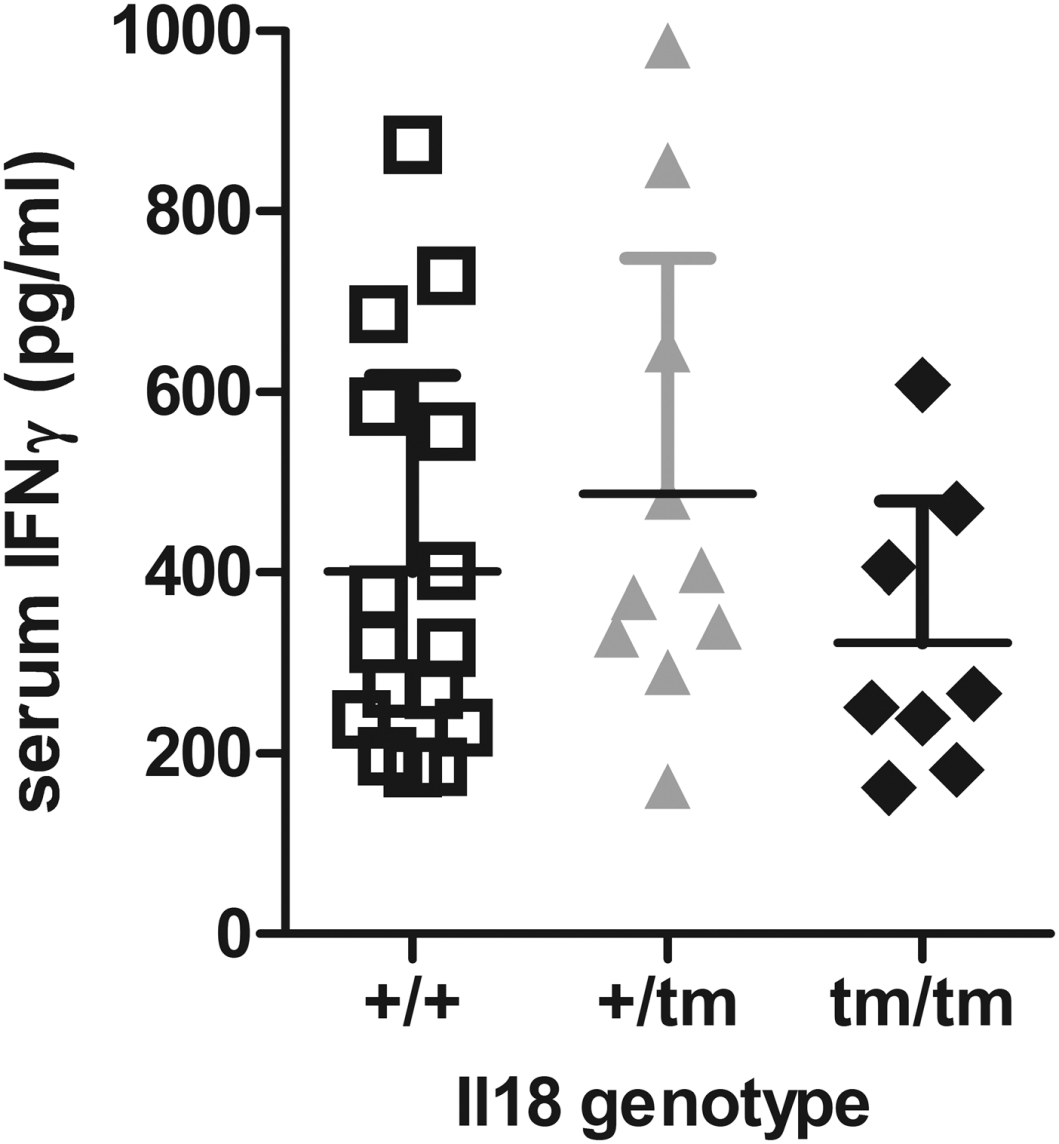
Deletion of IL-18 expression does not affect constitutive IFNγ production. Mice, aged between 11 and 35 weeks, were bled and IFNγ concentrations in sera were measured by ELISA. Presented are data from n = 15, 10, and 8 individuals of MRL^lpr^, MRL^lpr^Il18^+/tm^, and MRL^lpr^Il18^tm/tm^ mice, respectively, and the respective means +/- SD. The three data sets were analysed by ANOVA with Bonferroni’s post test and revealed no statistically significant differences.

IL-18, which promotes IFN-γ production [5] is constitutively expressed in *e*.*g*. macrophages and dendritic cells. Thus, we measured constitutive IFN-γ concentrations in sera, but, again, could not detect statistically significant differences depending on the IL-18 genotype (Fig 2). Differences were also absent when comparing age-matched subgroups of mice (not shown).

**Fig 3:**
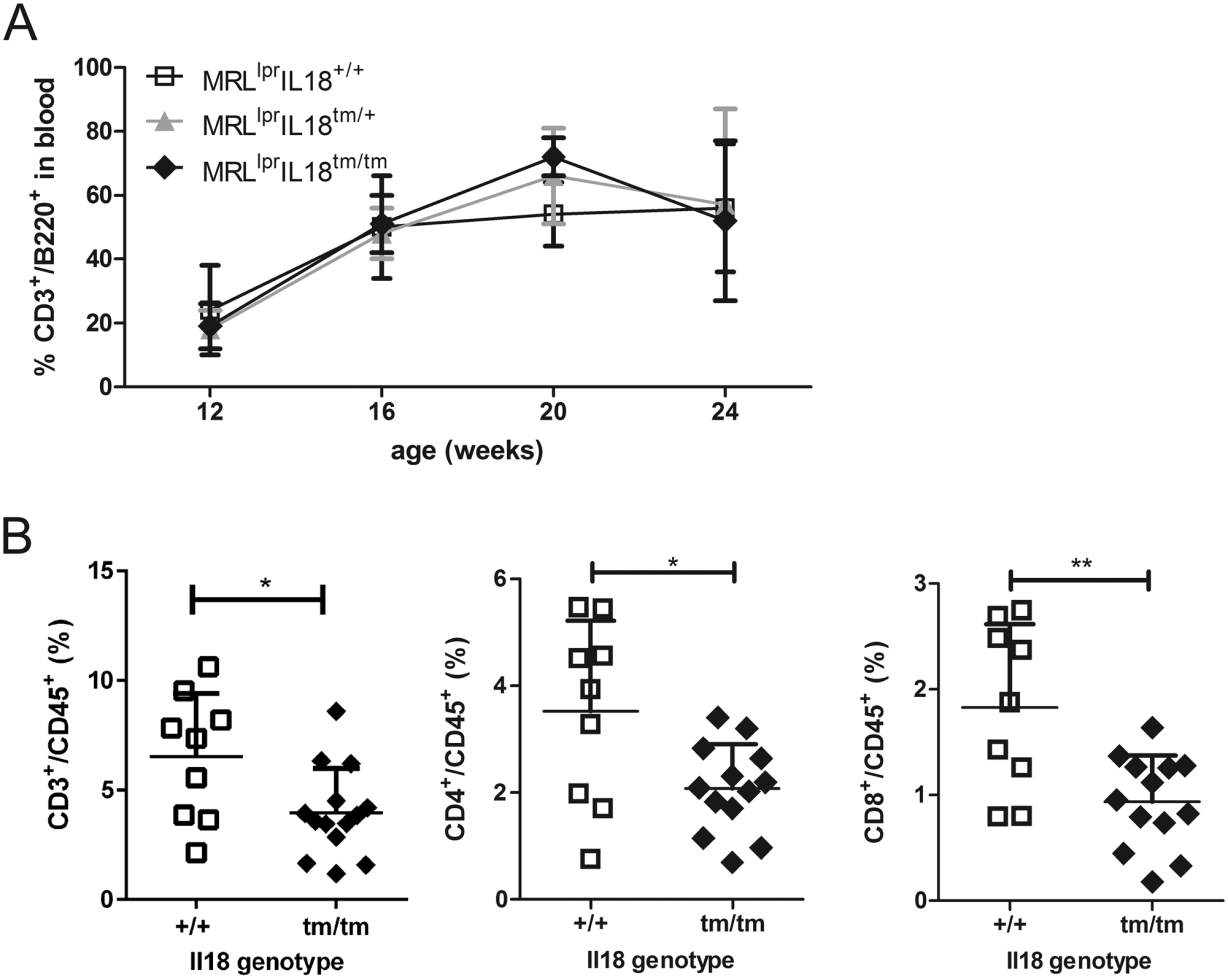
Flow cytometric analyses of T-cells. (A) MRL^lpr^, MRL^lpr^Il18^+/tm^, and MRL^lpr^Il18^tm/tm^ mice were bled and frequencies of blood cells were analysed using antibodies recognizing CD3 and B220. Presented are individual data from 3–23 evaluations and the respective means +/- SD. The three data sets were analysed by ANOVA with Bonferroni’s post test and revealed no statistically significant differences. (B) Single cell suspensions were prepared from kidneys of 19–29 week old MRL^lpr^ and MRL^lpr^Il18^tm/tm^ mice, and frequencies of CD3^++^, CD4^+^, and CD8^+^ cells within the CD45^+^ population were determined. Presented are data of individual evaluations and the respective means +/- SD. The data sets (n = 9 and 13, respectively) were analysed by Student’s T-test and revealed no statistically significant differences.

The lupus-like disease in MRL^lpr^ mice is paralleled by the persistence of CD3^+^B220^+^CD4^-^CD8^-^ cells, identified by the co-expression of CD3 and B220 and referred to as the DN T-cell population [34]. DN T-cells are numerously present in peripheral blood of MRL^lpr^ mice, however, in MRL^lpr^IL18^tm/tm^ mice their number is not statistically significantly different (Fig 3A). Also the other leukocyte subsets identified by the markers CD3, B220, CD4, and CD8 demonstrated no quantitative differences in peripheral blood between the mouse strains (data not shown).

The kidney is one of the mainly affected organs in the lupus-like disease of MRL^lpr^ mice [17]. Single cell suspensions prepared from kidneys of MRL^lpr^ and MRL^lpr^IL18^tm/tm^ mice consisted of about 10% CD45^+^ leukocytes. Within these, DN T-cells were absent, while the subsets identified by Ly-6G/C, CD11b, and F4/80 were readily detectable, however, without statistically significant quantitative differences between MRL^lpr+^ and MRL^lpr^IL18^tm/tm^ mice (not shown). In contrast, CD3^+^B220^-^ T-cells, both CD4^+^ and CD8^+^, were less frequent in the kidney cell suspensions of MRL^lpr^IL18^tm/tm^ mice as compared to that of MRL^lpr^ mice (Fig 3B).

**Fig 4:**
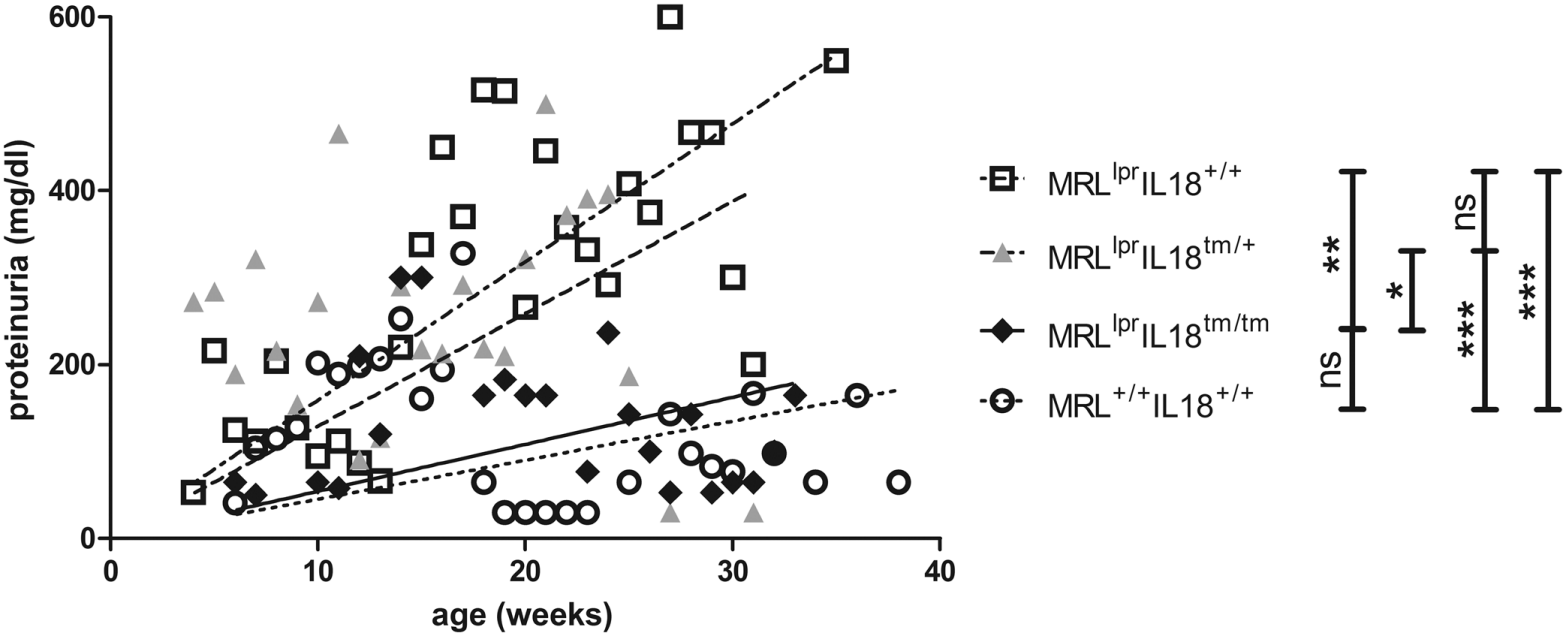
Deletion of IL-18 expression reduces proteinuria. Protein concentrations in spontaneously voided urine from MRL^lpr^, MRL^lpr^Il18^+/tm^, MRL^lpr^Il18^tm/tm^, and MRL^+/+^ mice were measured in weekly intervals. Within each strain, mice were grouped according to their age on a weekly base. Each symbol represents the mean of at least two individual evaluations, performed using a total of 13–39 individuals per strain. Lines show the linear regressions of the data of each strain. Statistical differences between the transformed data of each strain were analysed by ANOVA Kruskal Wallis test with Dunn’s post test (ns, not significant; *, p < 0.05; **, p < 0.01; ***, p < 0.005).

Since we found CD3^+^ T-cells less frequently in the kidneys of MRL^lpr^ mice due to the absence of IL-18, we analyzed urine protein concentration as a measure for functional kidney integrity in MRL^lpr^, MRL^lpr^IL18^+/tm^, and MRL^lpr^IL18^tm/tm^ mice. As a further control, in this experiment we also included wild type MRL (MRL^+/+^) mice, lacking the disease accelerating *lpr* mutation. While in MRL^+/+^ mice the progression of proteinuria was rather slow, it was significantly faster in MRL^lpr^ mice (Fig 4). In heterozygous mice (MRL^lpr^IL18^+/tm^) protein concentrations in urine increased as fast as in MRL^lpr^ mice. In MRL^lpr^IL18^tm/tm^ mice, in contrast, the kinetics of proteinuria development were significantly slower, virtually resembling that observed in MRL^+/+^ mice (Fig 4).

**Fig 5:**
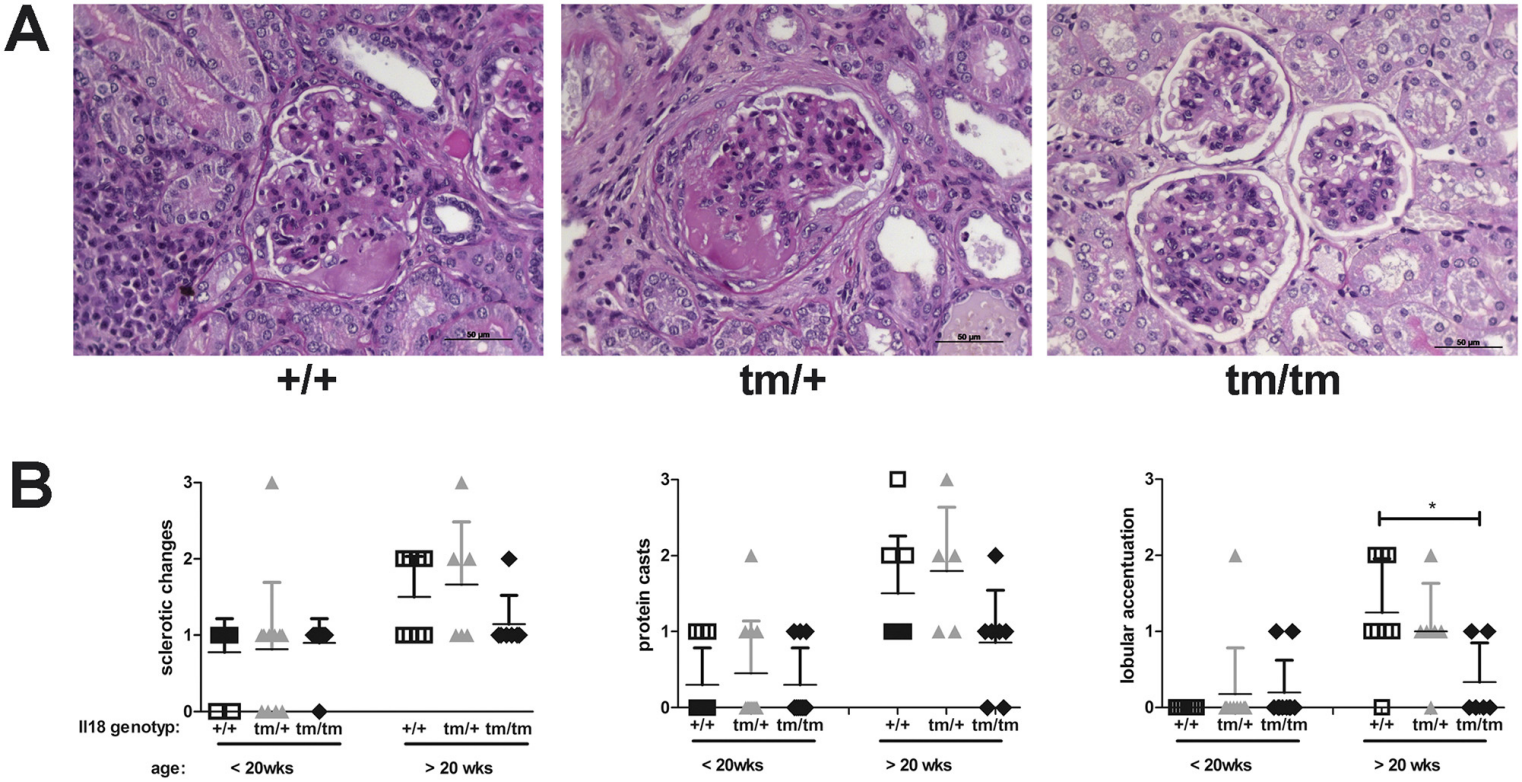
Histological analyses of kidney sections. Kidneys of MRL^lpr^, MRL^lpr^Il18^+/tm^, and MRL^lpr^Il18^tm/tm^ mice were removed, processed, and histologically analysed. (A) Presented are representative pictures of PAS-stained kidney sections obtained from MRL^lpr^ (+/+), MRL^lpr^Il18^+/tm^, (+/tm), and MRL^lpr^Il18^tm/tm^ (tm/tm) mice. (B) Quantitative evaluations of the sections were performed using the scoring system as detailed in Table 2. Presented are individual data for three evaluated parameters from 5–11 mice per strain and the respective means +/- SD. Differences between the three strains were analysed by ANOVA with Bonferroni’s post test (*, p < 0.05; no indication: no significance).

Next, we evaluated the kidneys histologically, both by light (Fig 5A) and by electron microscopy (data not shown). Kidney sections obtained from young (< 20 weeks) and old (> 20 weeks) MRL^lpr^, MRL^lpr^IL18^+/tm^, and MRL^lpr^IL18^tm/tm^ mice (chosen independently of their health status) were analyzed for several aspects as detailed in the materials and methods section. Of these, sclerotic changes of the mesangium, protein casts in distal tubuli, and lobular accentuation of glomerulopathy increased age-dependently in MRL^lpr^ and MRL^lpr^IL18^+/tm^ mice (Fig 5B). Importantly, while sclerotic changes of the mesangium and protein casts in distal tubuli increased age-dependently also in MRL^lpr^IL18^tm/tm^ mice, the lobular accentuation of glomerulopathy was significantly reduced in old MRL^lpr^IL18^tm/tm^ as compared to old MRL^lpr^ mice (Fig 5B).

## Discussion

Several studies point to a contribution of IL-18 to symptoms associated with human lupus and the lupus-like disease in mouse models [7, 35–38]. In the MRL^lpr^ mouse model resembling human SLE such evidence based on a genomic approach is discussed very controversially [29, 30]. These studies, in order to eliminate IL-18 bioactivity, deleted the IL-18 receptor, but more recently it has also been reported that the IL-18 receptor is necessary not only for IL-18 bioactivity but also for a yet not identified ligand [31], probably similar to the human IL-18 receptor ligand IL-37 [39]. Thus, in order to specifically analyze the function of IL-18 in murine SLE, in the present study, we deleted the gene encoding IL-18 in MRL^lpr^ mice and compared pathological and immunological manifestations with those of IL-18-competent MRL^lpr^ mice.

In MRL^lpr^ mice serum concentrations of IFN-γ are enhanced in comparison to not autoimmune-prone mouse strains and IFN signaling is essential for lupus development [20–23]. Blockade of IFN-γ signaling by deleting expression of the cytokine itself or its receptor results in reduced double-stranded DNA-directed autoantibody production and prolonged survival. Interestingly, lymphadenopathy and the number of DN T-cells are reduced in MRL^lpr^ only due to IFN-γ-deficiency, while it is unaffected by the lack of the IFN-γ receptor. IL-18 promotes IFN-γ expression in T-cells and NK cells [1], however, since IL-18-deficiency in MRL^lpr^ mice does not reduce IFN-γ concentration in blood, the constitutively enhanced production of IFN-γ is not a consequence of IL-18 function [25]. Probably due to the unaffected IFN-γ concentration, IL-18 deficiency in MRL^lpr^ mice affects neither survival nor the accumulation of DN T-cells accompanied by lymphadenopathy and splenomegaly. These data are in contrast to our previous ones obtained by a cDNA vaccination technique [27], probably reflecting that vaccination of MRL^lpr^ mice with an IL18-encoding plasmid not only led to the reduction of IL-18 bioactivity but also to additional beneficial effects, e.g. TLR9 activation [40, 41], leading to an amelioration of clinical parameters.

Thus, the effect of IL-18 in MRL^lpr^ mice seems to be restricted to the kidney inflammation and probably other organs as well, which we have not analyzed in this study. Such a specific function for IL-18 in autoimmune lupus nephritis as well as in other kidney diseases already has been proposed previously [39, 42–44]. Interestingly, deletion of IL-18 expression in MRL^lpr^ mice selectively reduced the intra-renal frequency of T-cells, both CD4^+^ and CD8^+^, while other leukocyte populations, such as granulocytes, monocytes, and macrophages were unaffected. This is in contrast to data provided by an ‘intra-renal IL-18 overexpression’ model [43], and may reflect an IL-18 concentration exceeding the physiologic range in the latter study.

Using a flow cytometric approach, we could not detect infiltrating DN T-cells, identified by the co-expression of CD3 and B220, in the kidneys of MRL^lpr^ mice, aged up to 4 months, while in cell suspensions obtained from spleens they were readily detectable (not shown). This result is in direct contradiction to the histological observation of CD3^+^IL-17^+^ cells in the kidneys of 5 months old MRL^lpr^ mice, which are claimed to be of the DN T-cell population [19]. Unfortunately, in the latter publication the CD4^-^CD8^-^ phenotype of the kidney infiltrating CD3^+^IL-17^+^ cells was not documented, but deduced from analyses using peripheral CD3^+^IL-17^+^ cells.

Surprisingly, the fast worsening general health status, quantified as ‘survival’, is unaffected by deletion of IL-18 expression, although specific parameters of nephritis are ameliorated. This may be due to the fact that only a few specific kidney pathological parameters were reduced due to IL-18 deletion, while others and also the gross inflammatory pathology remained unaffected.

In summary, using a genetic approach, we provide evidence that IL-18 plays a minor role in the autoimmune disease in MRL^lpr^ mice, essentially confirming the data observed in IL-18-deficient lupus-prone C57Bl/6 mice [45]. In our model, IL-18 does not affect the lupus-like pathogenesis generally, but specifically some parameters of affected end-organs, *i*.*e*. the kidneys.

## References

[1] Novick D, Kim S, Kaplanski G, Dinarello CA. Interleukin-18, more than a Th1 cytokine. Semin Immunol. 2013;25(6):439–48. 10.1016/j.smim.2013.10.014 24275602

[2] Neumann D, Martin M. Interleukin-12 upregulates the IL-18R beta chain in BALB/c thymocytes. J Interf Cytok Res. 2001;21(8):635–42. 10.1089/10799900152547902 11559442

[3] Tomura M, Zhou XY, Maruo S, Ahn HJ, Hamaoka T, Okamura H, et al A critical role for IL-18 in the proliferation and activation of NK1.1^+^ CD3^-^ cells. J Immunol. 1998;160(10):4738–46. 9590219

[4] Tomura M, Maruo S, Mu J, Zhou XY, Ahn HJ, Hamaoka T, et al Differential capacities of CD4^+^, CD8^+^, and CD4^-^CD8^-^ T cell subsets to express IL-18 receptor and produce IFN-gamma in response to IL-18. J Immunol. 1998;160(8):3759–65. 9558078

[5] Robinson D, Shibuya K, Mui A, Zonin F, Murphy E, Sana T, et al IGIF does not drive Th1 development but synergizes with IL-12 for interferon-gamma production and activates IRAK and NFkappaB. Immunity. 1997;7(4):571–81. 9354477 10.1016/s1074-7613(00)80378-7

[6] Yoshimoto T, Takeda K, Tanaka T, Ohkusu K, Kashiwamura S, Okamura H, et al IL-12 up-regulates IL-18 receptor expression on T cells, Th1 cells, and B cells: synergism with IL-18 for IFN-gamma production. J Immunol. 1998;161(7):3400–7. 9759857

[7] Sedimbi SK, Hägglöf T, Karlsson MC. IL-18 in inflammatory and autoimmune disease. Cell Mol Life Sci. 2013;70(24):4795–808. 10.1007/s00018-013-1425-y 23892891 PMC11113411

[8] Dinarello CA, Novick D, Kim S, Kaplanski G. Interleukin-18 and IL-18 binding protein. Front Immunol. 2013;4:289 10.3389/fimmu.2013.00289 24115947 PMC3792554

[9] Shimizu C, Fujita T, Fuke Y, Ito K, Satomura A, Matsumoto K, et al High circulating levels of interleukin-18 binding protein indicate the severity of glomerular involvement in systemic lupus erythematosus. Mod Rheumatol. 2012;22(1):73–9. 10.1007/s10165-011-0471-2 21656327

[10] Tsao BP. The genetics of human systemic lupus erythematosus. Trends Immunol. 2003;24(11):595–602. 14596884 10.1016/j.it.2003.09.006

[11] Chen S, Jiang F, Ren J, Liu J, Meng W. Association of IL-18 polymorphisms with rheumatoid arthritis and systemic lupus erythematosus in Asian populations: a meta-analysis. BMC Med Genet. 2012;13:107 10.1186/1471-2350-13-107 23153245 PMC3523015

[12] Song GG, Choi SJ, Ji JD, Lee YH. Association between interleukin-18 polymorphisms and systemic lupus erythematosus: a meta-analysis. Mol Biol Rep. 2013;40(3):2581–7. 10.1007/s11033-012-2344-y 23238919

[13] Wen D, Liu J, Du X, Dong JZ, Ma CS. Association of interleukin-18 (-137G/C) polymorphism with rheumatoid arthritis and systemic lupus erythematosus: a meta-analysis. Int Rev Immunol. 2014;33(1):34–44. 10.3109/08830185.2013.816699 23914907

[14] Wong CK, Li EK, Ho CY, Lam CW. Elevation of plasma interleukin-18 concentration is correlated with disease activity in systemic lupus erythematosus. Rheumatology (Oxford). 2000;39(10):1078–81. 11035126 10.1093/rheumatology/39.10.1078

[15] Amerio P, Frezzolini A, Abeni D, Teofoli P, Girardelli CR, De Pità O, et al Increased IL-18 in patients with systemic lupus erythematosus: relations with Th-1, Th-2, pro-inflammatory cytokines and disease activity. IL-18 is a marker of disease activity but does not correlate with pro-inflammatory cytokines. Clin Exp Rheumatol. 2002;20(4):535–8. 12175109

[16] Park MC, Park YB, Lee SK. Elevated interleukin-18 levels correlated with disease activity in systemic lupus erythematosus. Clin Rheumatol. 2004;23(3):225–9. 15168150 10.1007/s10067-004-0867-x

[17] Andrews BS, Eisenberg RA, Theofilopoulos AN, Izui S, Wilson CB, McConahey PJ, et al Spontaneous murine lupus-like syndromes. Clinical and immunopathological manifestations in several strains. J Exp Med. 1978;148(5):1198–215. 309911 10.1084/jem.148.5.1198 PMC2185049

[18] Bossu P, Singer GG, Andres P, Ettinger R, Marshak-Rothstein A, Abbas AK. Mature CD4^+^ T lymphocytes from MRL/lpr mice are resistant to receptor-mediated tolerance and apoptosis. J Immunol. 1993;151(12):7233–9. 7903104

[19] Zhang Z, Kyttaris VC, Tsokos GC. The role of IL-23/IL-17 axis in lupus nephritis. J Immunol. 2009;183(5):3160–9. 10.4049/jimmunol.0900385 19657089 PMC2766304

[20] Peng SL, Moslehi J, Craft J. Roles of interferon-gamma and interleukin-4 in murine lupus. J Clin Invest. 1997;99(8):1936–46. 9109438 10.1172/JCI119361 PMC508018

[21] Balomenos D, Rumold R, Theofilopoulos AN. Interferon-gamma is required for lupus-like disease and lymphoaccumulation in MRL-lpr mice. J Clin Invest. 1998;101(2):364–71. 9435308 10.1172/JCI750 PMC508575

[22] Haas C, Ryffel B, Le Hir M. IFN-gamma is essential for the development of autoimmune glomerulonephritis in MRL/Ipr mice. J Immunol. 1997;158(11):5484–91. 9164971

[23] Schwarting A, Wada T, Kinoshita K, Tesch G, Kelley VR. IFN-gamma receptor signaling is essential for the initiation, acceleration, and destruction of autoimmune kidney disease in MRL-Fas(lpr) mice. J Immunol. 1998;161(1):494–503. 9647261

[24] Nicoletti F, Di Marco R, Zaccone P, Xiang M, Magro G, Grasso S, et al Dichotomic effects of IFN-gamma on the development of systemic lupus erythematosus-like syndrome in MRL-lpr / lpr mice. Eur J Immunol. 2000;30(2):438–47. 10671199 10.1002/1521-4141(200002)30:2<438::AID-IMMU438>3.0.CO;2-D

[25] Neumann D, Del Giudice E, Ciaramella A, Boraschi D, Bossu P. Lymphocytes from autoimmune MRL lpr/lpr mice are hyperresponsive to IL-18 and overexpress the IL-18 receptor accessory chain. Journal of Immunology. 2001;166(6):3757–62. 10.4049/jimmunol.166.6.3757 11238617

[26] Esfandiari E, McInnes IB, Lindop G, Huang FP, Field M, Komai-Koma M, et al A proinflammatory role of IL-18 in the development of spontaneous autoimmune disease. J Immunol. 2001;167(9):5338–47. 11673550 10.4049/jimmunol.167.9.5338

[27] Bossu P, Neumann D, Del Giudice E, Ciaramella A, Gloaguen I, Fantuzzi G, et al IL-18 cDNA vaccination protects mice from spontaneous lupus-like autoimmune disease. Proc Natl Acad Sci USA. 2003;100(24):14181–6. 14615579 10.1073/pnas.2336094100 PMC283566

[28] Neumann D, Tschernig T, Popa D, Schmiedl A, de Lema GP, Resch K, et al Injection of IL-12-and IL-18-encoding plasmids ameliorates the autoimmune pathology of MRL/Mp-*Tnfrsf6* ^*lpr*^ mice: synergistic effect on autoimmune symptoms. Int Immunol. 2006;18(12):1779–87. 17077176 10.1093/intimm/dxl112

[29] Lin L, Peng SL. Interleukin-18 receptor signaling is not required for autoantibody production and end-organ disease in murine lupus. Arthritis Rheum. 2005;52(3):984–6. 15751069 10.1002/art.20961

[30] Kinoshita K, Yamagata T, Nozaki Y, Sugiyama M, Ikoma S, Funauchi M, et al Blockade of IL-18 receptor signaling delays the onset of autoimmune disease in MRL-Faslpr mice. J Immunol. 2004;173(8):5312–8. 15470078 10.4049/jimmunol.173.8.5312

[31] Gutcher I, Urich E, Wolter K, Prinz M, Becher B. Interleukin 18-independent engagement of interleukin 18 receptor-alpha is required for autoimmune inflammation. Nat Immunol. 2006;7(9):946–53. 16906165 10.1038/ni1377

[32] Takeda K, Tsutsui H, Yoshimoto T, Adachi O, Yoshida N, Kishimoto T, et al Defective NK cell activity and Th1 response in IL-18-deficient mice. Immunity. 1998;8(3):383–90. 9529155 10.1016/s1074-7613(00)80543-9

[33] Montgomery CA. Control of animal pain and distress in cancer and toxicologic research. J Am Vet Med Assoc. 1987;191(10):1277–81. 3692969

[34] Ohteki T, Seki S, Abo T, Kumagai K. Liver is a possible site for the proliferation of abnormal CD3^+^4^-^8^-^ double-negative lymphocytes in autoimmune MRL-lpr/lpr mice. J Exp Med. 1990;172(1):7–12. 2141631 10.1084/jem.172.1.7 PMC2188149

[35] Yap DY, Lai KN. The role of cytokines in the pathogenesis of systemic lupus erythematosus—from bench to bedside. Nephrology (Carlton). 2013;18(4):243–55. 23452295 10.1111/nep.12047

[36] Clark DN, Markham JL, Sloan CS, Poole BD. Cytokine inhibition as a strategy for treating systemic lupus erythematosus. Clin Immunol. 2013;148(3):335–43. 10.1016/j.clim.2012.11.001 23200699

[37] Kahlenberg JM, Kaplan MJ. The inflammasome and lupus: another innate immune mechanism contributing to disease pathogenesis? Curr Opin Rheumatol. 2014;26(5):475–81. 10.1097/BOR.0000000000000088 24992143 PMC4153426

[38] Ghoreishi M, Dutz JP. Cutaneous lupus erythematosus: recent lessons from animal models. Lupus. 2010;19(9):1029–35. 10.1177/0961203310370045 20693196

[39] Yang Y, Zhang ZX, Lian D, Haig A, Bhattacharjee RN, Jevnikar AM. IL-37 inhibits IL-18-induced tubular epithelial cell expression of pro-inflammatory cytokines and renal ischemia-reperfusion injury. Kidney Int. 2014 10.1038/ki.2014.295 25207880

[40] Christensen SR, Kashgarian M, Alexopoulou L, Flavell RA, Akira S, Shlomchik MJ. Toll-like receptor 9 controls anti-DNA autoantibody production in murine lupus. J Exp Med. 2005;202(2):321–31. 16027240 10.1084/jem.20050338 PMC2212997

[41] Christensen SR, Shupe J, Nickerson K, Kashgarian M, Flavell RA, Shlomchik MJ. Toll-like receptor 7 and TLR9 dictate autoantibody specificity and have opposing inflammatory and regulatory roles in a murine model of lupus. Immunity. 2006;25(3):417–28. 16973389 10.1016/j.immuni.2006.07.013

[42] Faust J, Menke J, Kriegsmann J, Kelley VR, Mayet WJ, Galle PR, et al Correlation of renal tubular epithelial cell-derived interleukin-18 up-regulation with disease activity in MRL-Faslpr mice with autoimmune lupus nephritis. Arthritis Rheum. 2002;46(11):3083–95. 12428253 10.1002/art.10563

[43] Menke J, Bork T, Kutska B, Byrne KT, Blanfeld M, Relle M, et al Targeting transcription factor Stat4 uncovers a role for interleukin-18 in the pathogenesis of severe lupus nephritis in mice. Kidney Int. 2011;79(4):452–63. 10.1038/ki.2010.438 20980973 PMC3197226

[44] He Z, Lu L, Altmann C, Hoke TS, Ljubanovic D, Jani A, et al Interleukin-18 binding protein transgenic mice are protected against ischemic acute kidney injury. Am J Physiol Renal Physiol. 2008;295(5):F1414–21. 10.1152/ajprenal.90288.2008 18753296 PMC2584896

[45] Lech M, Lorenz G, Kulkarni OP, Grosser MO, Stigrot N, Darisipudi MN, et al NLRP3 and ASC suppress lupus-like autoimmunity by driving the immunosuppressive effects of TGF-β receptor signalling. Ann Rheum Dis. 2014; 10.1136/annrheumdis-2014-205496 25135254

